# Components from the Leaves and Twigs of Mangrove *Lumnitzera racemosa* with Anti-Angiogenic and Anti-Inflammatory Effects

**DOI:** 10.3390/md16110404

**Published:** 2018-10-25

**Authors:** Szu-Yin Yu, Shih-Wei Wang, Tsong-Long Hwang, Bai-Luh Wei, Chien-Jung Su, Fang-Rong Chang, Yuan-Bin Cheng

**Affiliations:** 1 Graduate Institute of Natural Products, College of Pharmacy, Kaohsiung Medical University, Kaohsiung 807, Taiwan; s91412232@gmail.com (S.-Y.Y.); shihwei@mmc.edu.tw (S.-W.W.); scj820826@gmail.com (C.-J.S.); 2 Department of Medicine, Mackay Medical College, New Taipei City 252, Taiwan; 3 Graduate Institute of Natural Products, College of Medicine, and Chinese Herbal Medicine Research Team, Healthy Aging Research Center, Chang Gung University, Taoyuan 333, Taiwan; htl@mail.cgu.edu.tw; 4 Research Center for Chinese Herbal Medicine, Research Center for Food and Cosmetic Safety, and Graduate Institute of Health Industry Technology, College of Human Ecology, Chang Gung University of Science and Technology, Taoyuan 333, Taiwan; 5 Department of Anesthesiology, Chang Gung Memorial Hospital, Taoyuan 333, Taiwan; 6 Department of Life Science, National Taitung University, Taitung 950, Taiwan; blwei@nttu.edu.tw; 7 National Research Institute of Chinese Medicine, Ministry of Health and Welfare, Taipei 112, Taiwan; 8 Department of Medical Research, Kaohsiung Medical University Hospital, Kaohsiung 807, Taiwan

**Keywords:** neolignan, *Lumnitzera racemosa*, anti-angiogenesis, anti-inflammation

## Abstract

One new neolignan, racelactone A (**1**), together with seven known compounds (**2**−**8**) were isolated from the methanolic extract of the leaves and twigs of *Lumnitzera racemosa*. The structure of racelactone A (**1**) was determined on the basis of the mass and NMR spectroscopic data interpretation. With respect to bioactivity, compound 1 displayed an anti-angiogenic effect by suppressing tube formation. Furthermore, compounds **1**, **4**, and **5** showed significant anti-inflammatory effects with IC_50_ values of 4.95 ± 0.89, 1.95 ± 0.40, and 2.57 ± 0.23 *μ*M, respectively. The plausible biosynthesis pathway of racelactone A (**1**) was proposed.

## 1. Introduction

Mangroves are unique plants growing in the intertidal zone of the tropical and subtropical climates; these species are salt-tolerant and have mechanisms which affect a variety of cellular metabolic processes [1]. Plants of the mangrove genus *Lumnitzera* (Combretaceae) comprise more than 600 species in Asia, Austria, and Africa. Among them, *Lumnitzera racemosa* Willd. is native to the seashore of southern Taiwan. *L*. *racemosa* can grow up to a five-meter height; its bark is dark brown and rough, the leaves are slightly concave top round, and the fruits are drupe and with an ellipsoid shape [2]. The woods of *L*. *racemosa* are hard and have a long shelf life; they can be used as building materials, equipment, or fuels. *L. racemosa* is also fantabulous nectar plant, while the leaves are edible to date [3]. Traditionally, the sap of this plant is used to treat cutaneous pruritus, herpes, scabies, and thrush [4]. The chemical constituents of *L*. *racemosa* are fatty acids, flavonoids, polyisoprenoid alcohols, tannins, and triterpenoids [5]. Pharmacological studies of the extracts from *L*. *racemosa* demonstrated antibacterial, antifungal, antihypertensive, antioxidant, cytotoxic, and hepatoprotective activities [6,7,8,9].

Inflammation is tightly associated with carcinogenesis and the disease progression of cancer [10]. Angiogenesis has been reported to facilitate the growth and dissemination of cancer cells in tumor microenvironments [11]. Plentiful studies reveal that inhibition of inflammation and angiogenesis is an effective therapeutic strategy to suppress cancer development and metastasis [12,13]. Our preliminary pharmacological investigation indicated that methanolic extract of the leaves and twigs of *L. racemosa* exerted promising anti-angiogenic and anti-inflammatory responses. Herein, we describe the isolation, structural elucidation, and bioactivities of one new neolignan, racelactone A (**1**), along with seven known compounds (**2**–**8**).

## 2. Results

**Figure 1:**
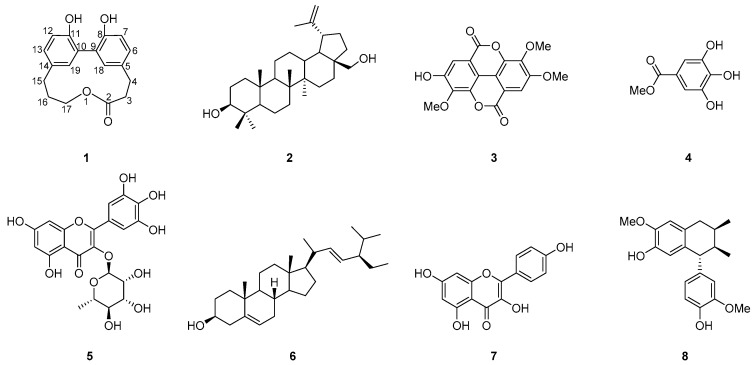
Structures of compounds **1**−**8**.

In the present study, the methanolic extracted from *L*. *racemosa* was partitioned with ethyl acetate (EtOAc) and water (H_2_O). The EtOAc layer was further partitioned with *n*-hexane and 75% methanol (MeOH) in H_2_O to give a 75% MeOH_(aq)_ layer. One new compound, racelactone A (**1**), and seven known compounds: Botulin (**2**) [14,15], 3,4,3′-tri-*O*-methyl ellagic acid (**3**) [16], methyl gallate (**4**) [17], myricitrin (**5**) [18], stigmasterol (**6**) [19], kaempferol (**7**) [20], and isoguaiacin (**8**) [21] were identified from the 75% MeOH_(aq)_ layer. The structures of compounds (**1**–**8**) are shown in Figure 1.

Racelactone A (**1**) was isolated as an amorphous powder, light yellow, having a molecular formula determined as C_18_H_18_O_4_. Ten indices of hydrogen deficiency were calculated, in accord with the high-resolution electrospray ionisation mass spectrometry (HRESIMS) data (*m*/*z* 321.10955 [M + Na]^+^) (Figure S1) and NMR spectrum. The IR spectrum (Figure S2) of **1** suggested the presence of hydroxy (3364 cm^−1^), carbonyl (1709 cm^−1^) and aromatic (1503, 1586 cm^−1^) functionalities. In ^1^H NMR (Figure S3), data revealed six olefinic methines (*δ*_H_ 6.81, 6.87, 7.01, 7.03, 7.06, and 7.18), and five methylenes (*δ*_H_ 2.25, 2.58, 2.82, 2.98, and 4.29) (Table 1). The ^13^C (Figure S4) and DEPT NMR spectrum (Table 1) of **1** noted eighteen carbon signals, including one carbonyl (*δ*c 175.0), six olefinic methines (*δ*c 113.3, 115.9, 116.4, 128.3, 129.2, and 133.1), six nonprotonated carbons (*δ*c 126.6, 127.2, 131.4, 132.7, 151.9, and 152.7), and five methylenes (*δ*c 25.2, 29.7, 30.7, 35.7, and 65.7). Analyses on a set of signals and coupling constants at *δ*_H_ 6.87 (d, 1H, *J* = 8.2 Hz, H-12), 7.03 (dd, 1H, *J* = 8.2, 2.4 Hz, H-13), and 7.06 (d, 1H, *J* = 2.5 Hz, H-18) as well as another set at *δ*_H_ 7.01 (dd, 1H, *J* = 8.2, 2.5 Hz, H-6), 6.81 (d, 1H, *J* = 8.1 Hz, H-7), and 7.19 (d, 1H, *J* = 2.5 Hz, H-19) led to the identification of two 1,3,4-trisubstituted phenyl moieties. From analyses of the NMR, UV (Figure S5), and IR data, compound **1** was determined to be a neolignan. Compound **1** showed similar ^1^H and ^13^C NMR signals (Table 1), partially similar to those of corniculatolide A, which has an ether bridge between two propylphenyl moieties [22,23]—except for the presence of two unusual quaternary carbon signals at *δ*c 126.6 and *δ*c 127.2 instead of two signals at *δ*c 149.0 and *δ*c 154.2 in corniculatolide A. This indicated a new carbon–carbon linkage formation in the target molecule.

**Figure 2:**
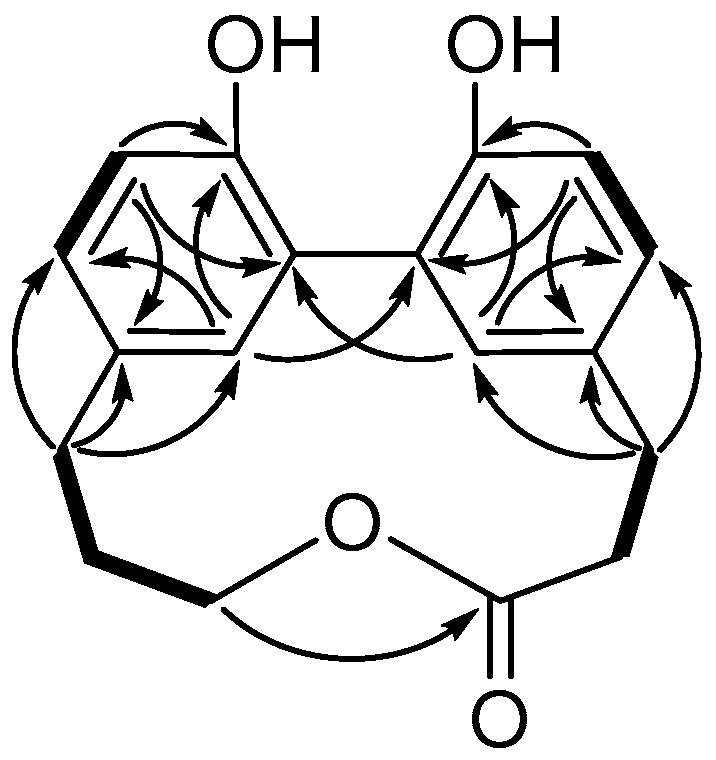
COSY (bold bond) and selected HMBC (arrow) correlations of **1**.

**Figure 3:**
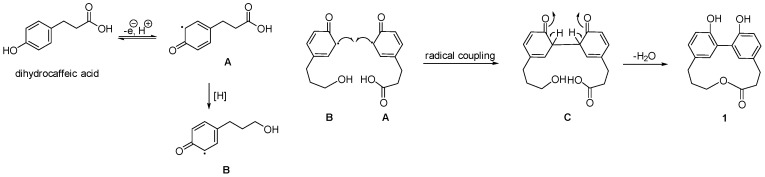
Plausible biosynthesis pathway of racelactone A (**1**).

The planar structure of **1** was established by the correlation spectroscopy (COSY) (Figure S6) and heter onuclear multiple bond correlation (HMBC) (Figure S7) correlations (Figure 2). The COSY correlations established the fragments of H-3/H-4, H-6/H-7, H-12/H-13, and H-15/H-16/H-17 of compound **1**. The HMBC correlations of H-4/C-5, C-6, and C-18 and the correlations of H-15/C-13, C-14, and C-19 determined the linkages of two sets of propyl and phenyl functions, respectively. The macroring connection system of two phenylpropanoid moieties was completed on the basis of a key HMBC correlation between H-17/C-2, H-18/C-10, and H-19/C-9. As mentioned above, compound **1** was categorized as a macrolactone, and named racelactone A. The biosynthesis of racelactone A is proposed to be initiated by a PAL enzyme of phenylalanine to form dihydrocaffeic acid. The precursor was resonated to form intermediates A and B. Phenoxy radicals of intermediates A and B were linked to generate the intermediate C, which was structured as racelactone A during a cyclization (Figure 3).

**Figure 4:**
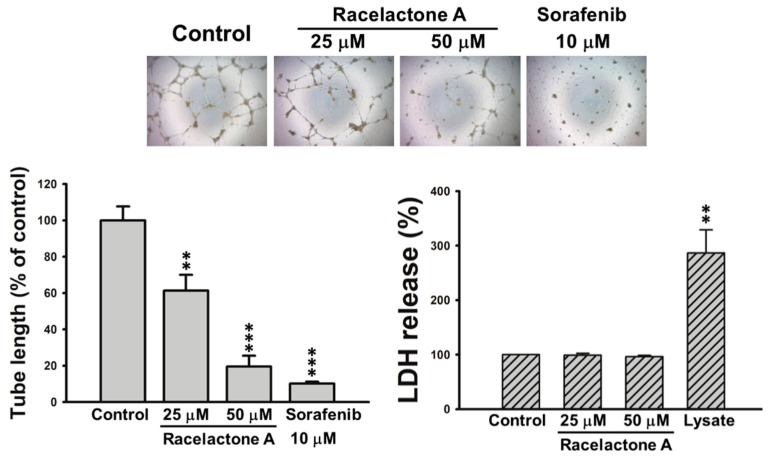
Effect of racelactone A (**1**) on tube formation and cytotoxicity of human endothelial progenitor cells (EPCs). EPCs were with the indicated concentrations of racelactone A and sorafenib for 24 h. The capillary-like structure formation and lactate dehydrogenase (LDH) release were determined by tube formation and cytotoxicity assay, respectively. Representative images of EPCs’ tube formation were shown (phase contrast, 40×). Data represent the mean ± SEM of five independent experiments. ** *p* < 0.01, *** *p* < 0.001 compared with the control group.

Circulating endothelial progenitor cells (EPCs) have been reported to promote tumor angiogenesis and metastasis [24,25]. Tumors can secrete a variety of angiogenic factors to induce the recruitment of EPCs from bone marrow to the tumor site. Recruited EPCs enter the circulation system from their niche in bone marrow and extravasate with the chemotactic stimuli. After reaching the tumor site, EPCs differentiate into the structural part of the tumor vasculature, which contributes to tumor progression. Furthermore, EPCs have the ability to release pro-inflammatory cytokines that facilitate the growth and metastatic spread of tumors. Compelling evidence suggests that selective targeting of EPCs represents a novel therapeutic strategy for cancer treatment [26]. The differentiation and formation of capillary vessels is the most critical process during EPCs angiogenesis. Therefore, we performed tube formation assay to evaluate the anti-angiogenic activity of racelactone A in EPCs. As shown in Figure 4, the capillary tube-like structure was suppressed by racelactone A in a concentration-dependent manner. Sorafenib, a well-known angiogenesis inhibitor, was used as the positive control. To confirm this anti-angiogenic effect was not caused by the potential cytotoxicity of racelactone A, the release of lactate dehydrogenase (LDH) was measured in racelactone A-treated EPCs. We found that no statistical difference was observed between the control EPCs and EPCs treated with racelactone A. Collectively, these results reveal that the anti-angiogenic effect of racelactone A is not due to the cytotoxic action in human EPCs.

All compounds were subjected to anti-inflammatory assays on superoxide anion generation and elastase release in fMLF/CB-induced human neutrophils inhibitory effects. Fortunately, the new compound **1** selectively displayed significant inhibitory activity on superoxide anion generation (IC_50_ = 4.95 ± 0.89 μM). The known compounds **4** and **5** also showed strong activity (Table 2).

## 3. Materials and Methods

### 3.1. General Experimental Procedures

Optical rotation was measured on a JASCO P-1020 digital polarimeter (Tokyo, Japan). UV data were recorded on a JASCO V-530 UV/VIS Spectrophotometer (Tokyo, Japan). High-resolution ESIMS data were obtained on a Bruker APEX II spectrometer (Billerica, MA, USA)). The IR spectrum was measured on a Perkin Elmer system 2000 FT-IR spectrophotometer (Waltham, MA, USA). The NMR spectra were obtained by JEOL JNM-ECS 400 MHz NMR (Akishima, Japan). Merck (Darmstadt, Germany) silica gel 60 and GE Healthcare (Chicago, IL, USA) Sephadex LH-20 were used for column chromatography. The instrumentation for HPLC was composed of a Shimadzu LC-10AD pump (Kyoto, Japan) and a Shimadzu SPD-M10A PDA detector.

### 3.2. Material

Specimens of *Lumnitzera racemosa* were collected in south Taiwan, in August 2015. The research samples were identified by Yuan-Bin Cheng. A voucher specimen (no. KMU-LR01) was deposited in the Graduate Institute of Natural Products, College of Pharmacy, Kaohsiung Medical University.

### 3.3. Extraction and Isolation

The air-dry twigs and leaves (15.0 kg) of *L. racemosa* were ground and extracted thrice with MeOH (40 L) at room temperature. The solvent was concentrated under reducing pressure to yield crude extracts. The MeOH crude extracts were partitioned between H_2_O/EtOAc (1:1) to afford two portions. The EtOAc part was partitioned with *n*-hexane and 75% MeOH in water (1:1). The 75% MeOH_(aq)_ layer (88.5 g) was subjected to a silica gel column stepwise eluted with *n*-hexane/EtOAc to yield botulin (221.1 mg), 3,4,3′-Tri-*O*-methyl ellagic acid (3330.0 mg), methyl gallate (4730.0 mg), myricitrin (5599.5 mg), and six fractions (A−J). Fraction C (830 mg) was isolated by silica gel column to give stigmasterol (63.4 mg). Fraction E (2.4 g) was chromatographed over a silica gel column to afford six subfractions (E.1−E.6). Subfraction E.4 (196.9 mg) was further separated by an LH-20 column, eluted with 100% MeOH to yield kaempferol (712.8 mg). Racelactone A (111.0 mg) and isoguaiacin (83.7 mg) were obtained from subfraction D.1 (144.7 mg) by LH-20 column, eluted with 100% MeOH and Phenyl-hexyl column (Luna phenyl-hexyl, 100 Å, 250 × 10 mm, Phenomenex®) stepwise from 70% to 100% MeOH_(aq)_.

Racelactone A (1): Light yellow amorphous powder; ${\left[\alpha \right]}_{D}^{26}$ ‒0.6 (*c* 0.05, MeOH); UV (MeOH) *λ*_max_ (log *ε*) 299 (2.85), 252 (2.95), 215 (3.34) nm; IR (neat) *v*_max_: 3364, 1709, 1503, 1411 cm^−1^; ^1^H NMR and ^13^C NMR data, see Table 1; HRESIMS *m*/*z* 321.10955 [M + Na]^+^ (calcd for C_18_H_18_O_4_Na^+^: 321.10973).

### 3.4. Preparation of Human EPCs

The ethical approval for the collection of human EPCs was granted by the Institutional Review Board of Mackay Medical College, New Taipei City, Taiwan (P1000002). Prior to collecting the peripheral blood from healthy donors, informed consent was acquired. After density centrifugation Ficoll-Paque plus (Amersham Biosciences, Uppala, Sweden), peripheral blood mononuclear cells (PBMCs) were isolated from the fractionated blood components. CD34-positive progenitor cells were isolated from PMBCs with CD34 MicroBead kit and MACS Cell Separation System (Miltenyi Biotec, Bergisch Gladbach, Germany). Further isolation and preservation of CD34-positive EPCs were performed as described previously [27,28]. In the present study, all experiments were carried out on EPCs between passages 10 and 18. EPCs were cultured with 1% gelatin-coated plasticware and MV2 complete medium (PromoCell, Heidelberg, Germany) with 20% defined fetal bovine serum (FBS) (HyClone, Logan, UT, USA) in humidified incubator containing 5% CO_2_ at 37 °C.

### 3.5. Tube Formation Assay

The capillary tube formation assay was carried out on Matrigel-coated 96-well plates. EPCs were seeded with the density of 1.25 × 10^4^ cells per well and incubated in an MV2 complete medium with 2% FBS and the indicated concentration of tested compounds for 24 h at 37 °C. Quantifications of EPCs differentiation and capillary-like tube formation were done with photomicrographs taken by an inverted phase contrast microscope. The long axis of each tube was measured with Image J software in 3 randomly chosen fields per well.

### 3.6. Cytotoxicity Assay

5 × 10^3^ of EPCs per well were seeded onto 96-well plates and incubated with an MV2 complete medium containing 2% FBS in the presence of vehicle (DMSO) or racelactone A. The quantification of LDH release in the medium was done with a cytotoxicity assay kit (Promega, Madison, WI, USA).

### 3.7. Superoxide Anion and Elastase Release Assays

The assay on superoxide anion generation and elastase release in response to fMLF stimulation of neutrophils were assayed by the same method as those of the reference published by co-author Professor Tsong-Long Hwang [29].

## 4. Conclusions

In summary, eight compounds, including one unusual macrolactone neolignan, were isolated and identified during a phytochemical investigation of the Taiwanese mangrove, *L. racemosa*. The new compound shows promising activities to anti-angiogenic and anti-inflammatory effects. Our findings suggest that racelactone A (**1**) may serve as a lead compound worthy of further development against angiogenesis-related diseases or inflammation-facilitated disorders, especially for the treatment of cancer.

## Figures and Tables

**Table 1:** ^1^H and ^13^C NMR data of **1***^a^* in acetone-*d_6_*.

Position	*δ*_H_, mult (*J* in Hz)	*δ*_C_, Type	HMBC (^1^H-^13^C)
2	-	175.0, C	-
3	2.58, m	35.7, CH_2_	2, 4, 5
4	2.98, m	29.7, CH_2_	2, 3, 5, 6, 18
5	-	132.7, C	-
6	7.01, dd (8.1, 2.5)	128.3, CH	7, 8, 18
7	6.81, d (8.1)	115.9, CH	5, 6, 8, 9
8	-	152.7, C	-
9	-	127.2, C	-
10	-	126.6, C	-
11	-	151.9, C	-
12	6.87, d (8.2)	116.4, CH	10, 11, 14
13	7.03, dd (8.2, 2.5)	129.2, CH	11, 12, 15, 19
14	-	131.4, C	-
15	2.82, m	30.7, CH_2_	13, 14, 16, 17, 19
16	2.25, m	25.2, CH_2_	14, 15, 17
17	4.29, t (5.0)	65.7, CH_2_	2, 15, 16
18	7.06, d (2.5)	133.1, CH	4, 6, 8, 10
19	7.19, d (2.5)	133.3, CH	9, 11, 13

*^a^*^1^H and ^13^C NMR data (*δ*) were measured at 400 and 100 MHz, respectively; chemical shifts are in ppm.

**Table 2:** Inhibitory effects of isolates on superoxide anion generation and elastase release in fMLF/CB-induced human neutrophils.

Compound	Percentage of IC_50_ (μM) *^a^*			
	Superoxide Anion		Elastase Release	
**1**	4.95 ± 0.89	**	>10	
**4**	1.95 ± 0.40	***	>10	
**5**	2.57 ± 0.23	***	>10	
**genistein** *^b^*	1.54 ± 0.37	***	17.47 ± 2.80	***

Percentage of inhibition (Inh %) at 10 μM concentration. Results are presented as mean ± SEM (n = 3~5). ** *p* < 0.01, *** *p* < 0.001 compared with the control. *^a^* Concentration necessary for 50% inhibition (IC_50_). *^b^* positive control.

## Supplementary Materials

The following are available online at http://www.mdpi.com/1660-3397/16/11/404/s1, Figure S1: HRESIMS of racelactone A (**1**). Figure S2: IR spectrum of racelactone A (**1**). Figure S3: ^1^H NMR Spectrum of racelactone A (**1**) in acetone-*d*_6_. Figure S4: ^13^C NMR Spectrum of racelactone A (**1**) in acetone-*d*_6_. Figure S5: UV spectrum of racelactone A (**1**). Figure S6: COSY Spectrum of racelactone A (**1**) in acetone-*d*_6_. Figure S7: HMBC Spectrum of racelactone A (**1**) in acetone-*d*_6_.
