# Supplementary material for: Components from the Leaves and Twigs of Mangrove Lumnitzera racemosa with Anti-Angiogenic and Anti-Inflammatory Effects
Source: Mar Drugs. 2018 Oct 25;16(11):404. doi: 10.3390/md16110404 (PMC6267291; doi:10.3390/md16110404)

Supplementary data

Components from the Leaves and Twigs of Mangrove *Lumnitzera racemosa* with Anti-Angiogenic and Anti-Inflammatory Effects

Szu-Yin Yu[1†], Shin-Wei Wang[1,2†], Tsong-Long Hwang[3,4,5], Bai-Luh Wei[6], Chien-Jung Su[1], Fang-Rong Chang[1,7,*], and Yuan-Bin Cheng[1,8,*]

[1] Graduate Institute of Natural Products, College of Pharmacy, Kaohsiung Medical University, Kaohsiung 807, Taiwan; aaronfrc@kmu.edu.tw (F.-R. C.); s91412232@gmail.com (S.-Y. Y.); shihwei@mmc.edu.tw (S.-W. W.); jmb@kmu.edu.tw (Y.-B. C.)
[2] Department of Medicine, Mackay Medical College, New Taipei City 252, Taiwan; shihwei@mmc.edu.tw (S.-W. W.)
[3] Graduate Institute of Natural Products, College of Medicine, Chang Gung University, Kweishan 333, Taoyuan, Taiwan; htl@mail.cgu.edu.tw (T.-L. H.)
[4] Research Center for Industry of Human Ecology, Research Center for Chinese Herbal Medicine, and Graduate Institute of Health Industry Technology, College of Human Ecology, Chang Gung University of Science and Technology, Taoyuan 333, Taiwan
[5] Department of Anesthesiology, Chang Gung Memorial Hospital, Taoyuan 333, Taiwan
[6] Department of Life Science, National Taitung University, Taitung 950, Taiwan; blwei@nttu.edu.tw (B.-L. W.)
[7] National Research Institute of Chinese Medicine, Ministry of Health and Welfare, Taipei 112, Taiwan
[8] Department of Medical Research, Kaohsiung Medical University Hospital, Kaohsiung 807, Taiwan
† These authors contributed equally to this work.

Table of Contents

Figure S1: HRESIMS of racelactone A (**1**).

Figure S2: IR spectrum of racelactone A (**1**).

Figure S3: ^{1}H NMR Spectrum of racelactone A (**1**) in acetone-*d*$_6$.

Figure S4: ^{13}C NMR Spectrum of racelactone A (**1**) in acetone-*d*$_6$.

Figure S5: UV spectrum of racelactone A (**1**).

Figure S6: COSY Spectrum of racelactone A (**1**) in acetone-*d*$_6$.

Figure S7: HMBC Spectrum of racelactone A (**1**) in acetone-*d*$_6$.

Figure S1: HRESIMS of racelactone A (**1**).

Mass Spectrum SmartFormula Report

Analysis Info

Analysis Name	D:\Data\g4\RLH_000006.d	12/26/2016 4:39:47 PM
Method	broadband first signal	Operator: YU HSIAO-CHING
Sample Name	RL-H	Instrument: BRUKER FT-MS solariX
Comment	ESI Positive	

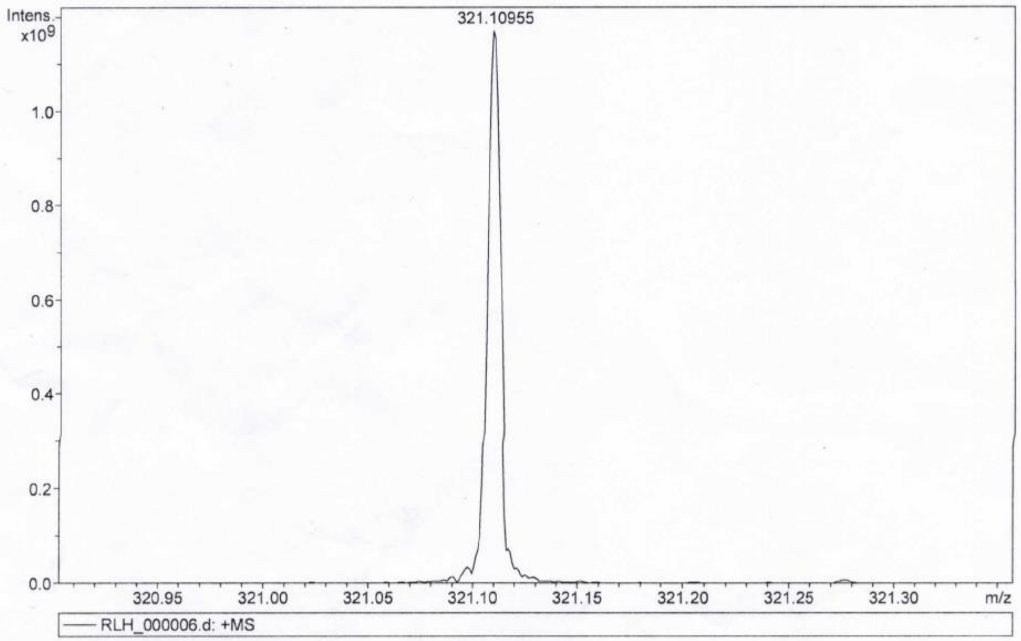

Meas. m/z	#	Formula	Score	m/z	err [mDa]	err [ppm]	mSigma	rdb	e⁻ Conf	N-Rule
321.10955	1	C 18 H 18 Na O 4	100.00	321.10973	0.18	0.57	5.7	9.5	even	ok

S2: IR spectrum of racelactone A (**1**).

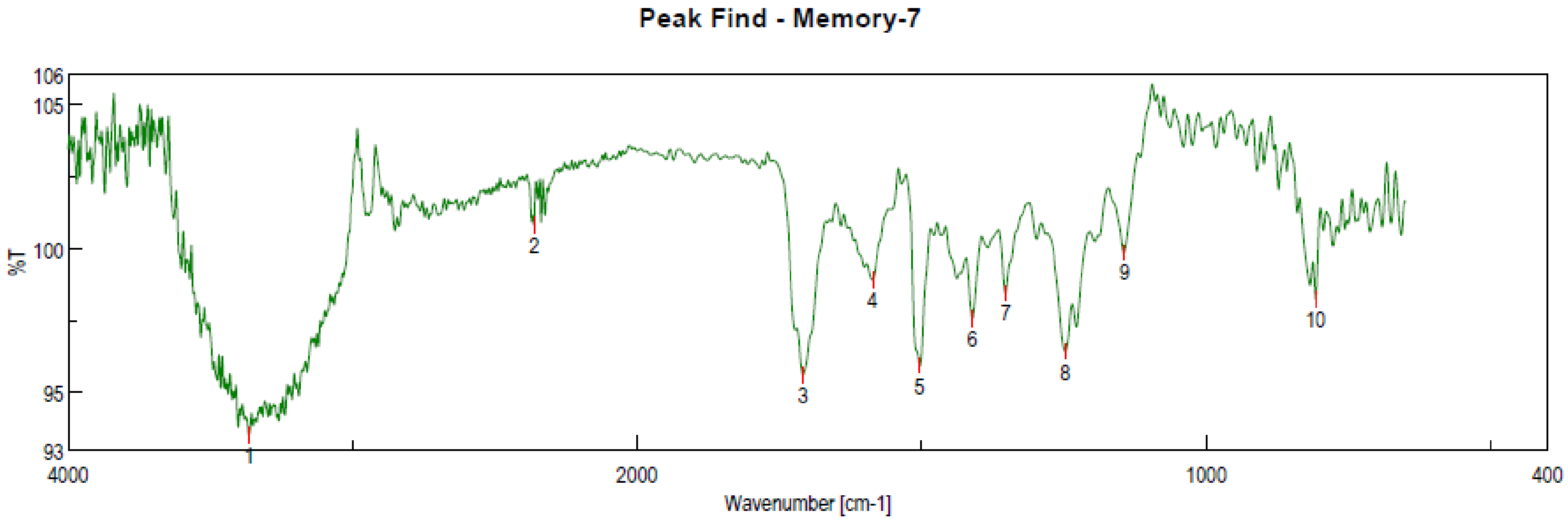

[Result of Peak Picking]

No.	Position	Intensity	No.	Position	Intensity	No.	Position	Intensity
1	3364.21	93.5209	2	2362.37	100.81	3	1708.62	95.605
4	1586.16	98.9065	5	1503.24	95.917	6	1410.67	97.5677
7	1351.86	98.4559	8	1247.72	96.4179	9	1143.58	99.8734
10	808.028	98.2354						

Figure S3: ^{1}H NMR Spectrum of racelactone A (**1**) in acetone-d_6.

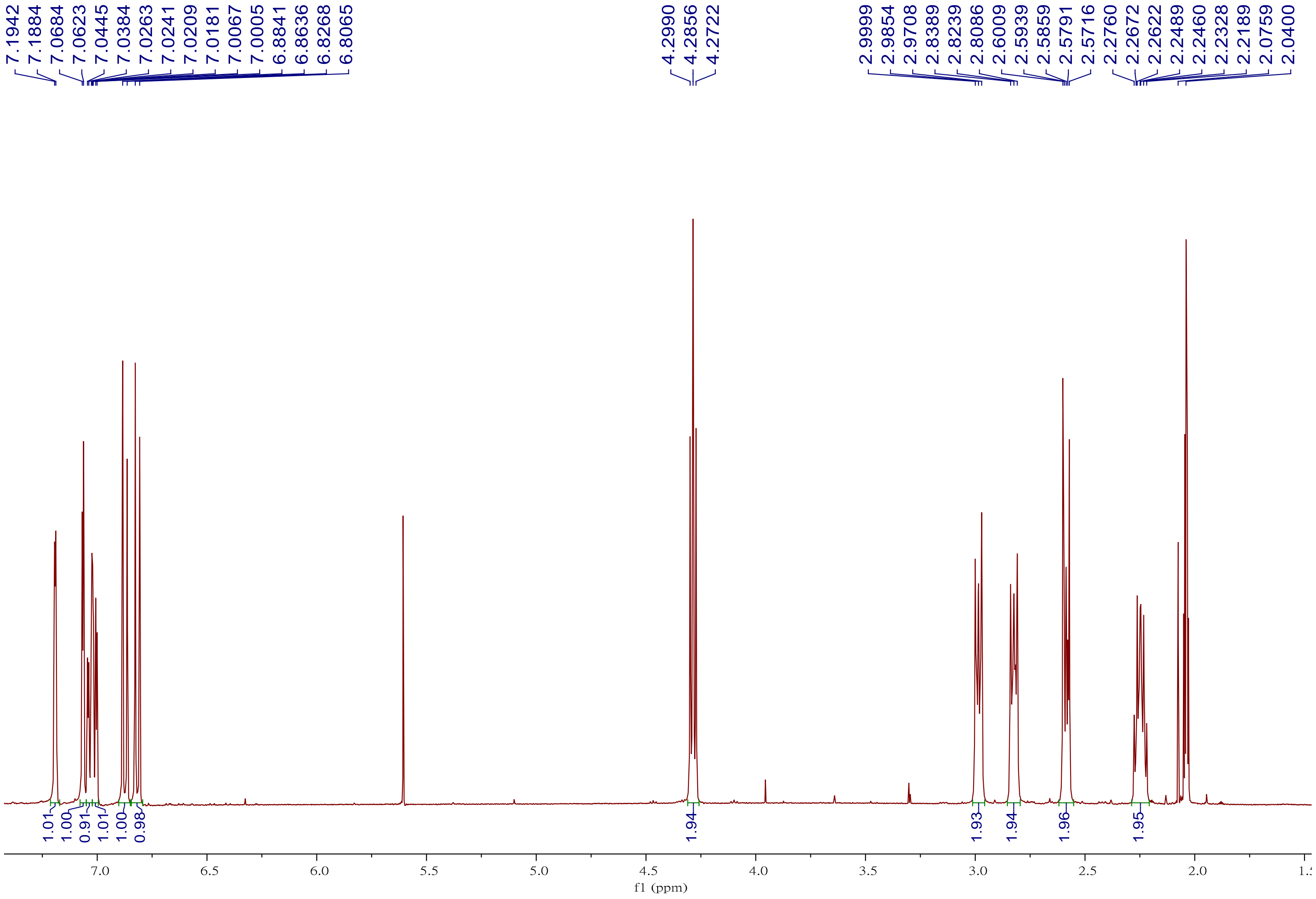

Figure S4: ^{13}C NMR Spectrum of racelactone A (**1**) in acetone-d_6.

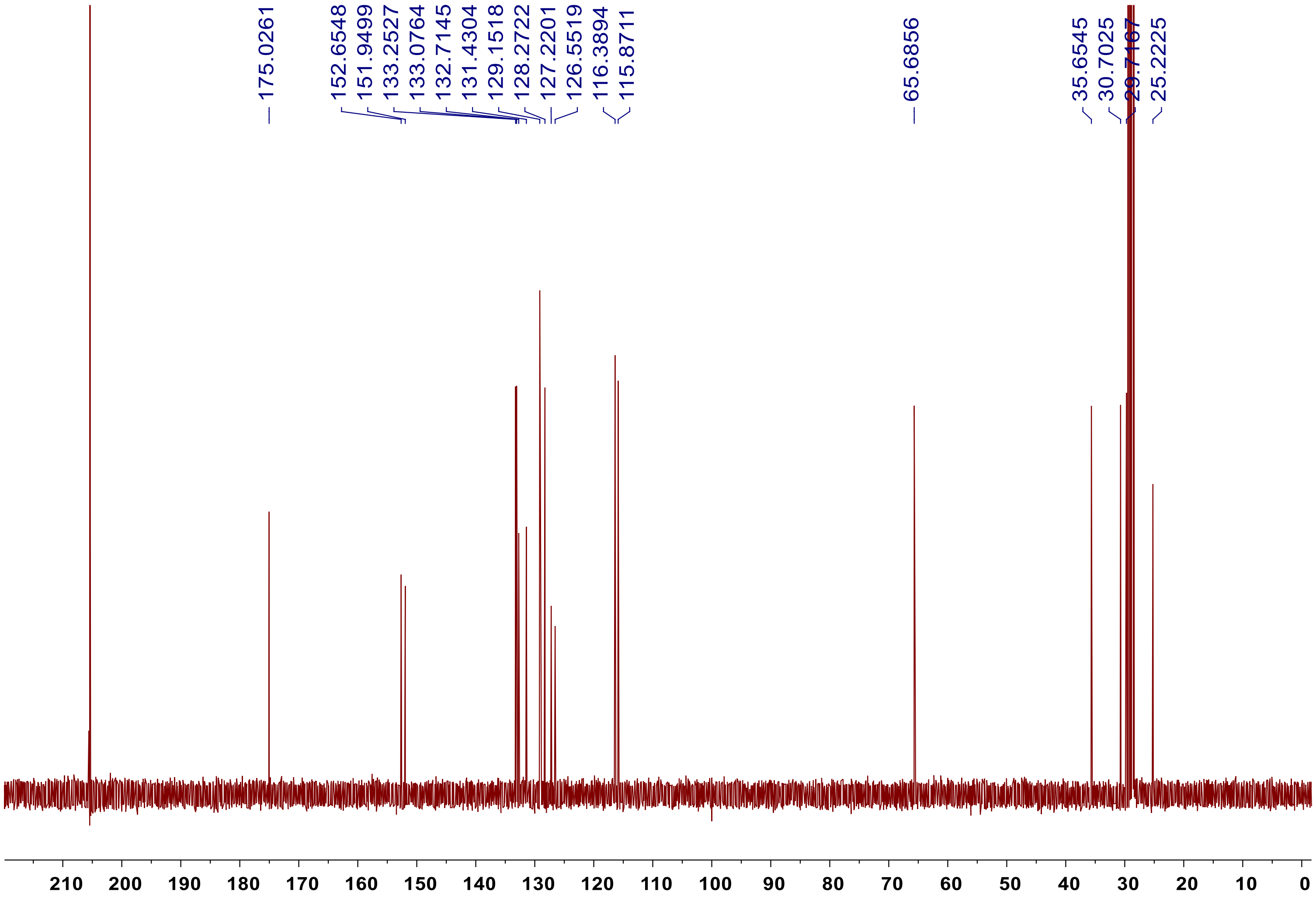

Figure S5: UV spectrum of racelactone A (**1**).

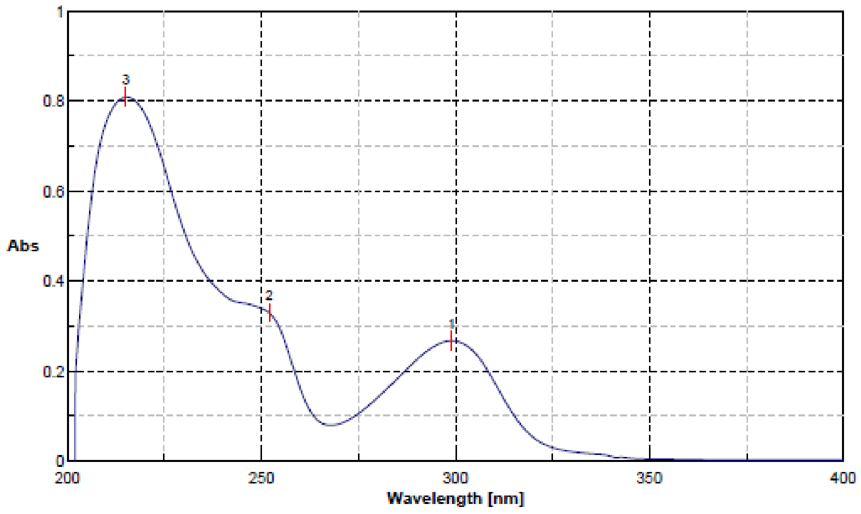

Date	2016/10/26 3:07下午
File name	Memory#2
Model	V-530
Serial No.	B114860512
Band width	2.0 nm
Response	Medium
Measurement range	400 - 200 nm
Data pitch	1nm
Scanning speed	200nm/min
Sample ID	701
No. of cycle	3
Cycle No.	3
Cycle interval	0 sec
Sample name	
Operator	KMU
Comment	

No.	nm	Abs	No.	nm	Abs	No.	nm	Abs
1	299	0.26688	2	252	0.32931	3	215	0.80899

Figure S6: COSY Spectrum of racelactone A (**1**) in acetone-*d*6.

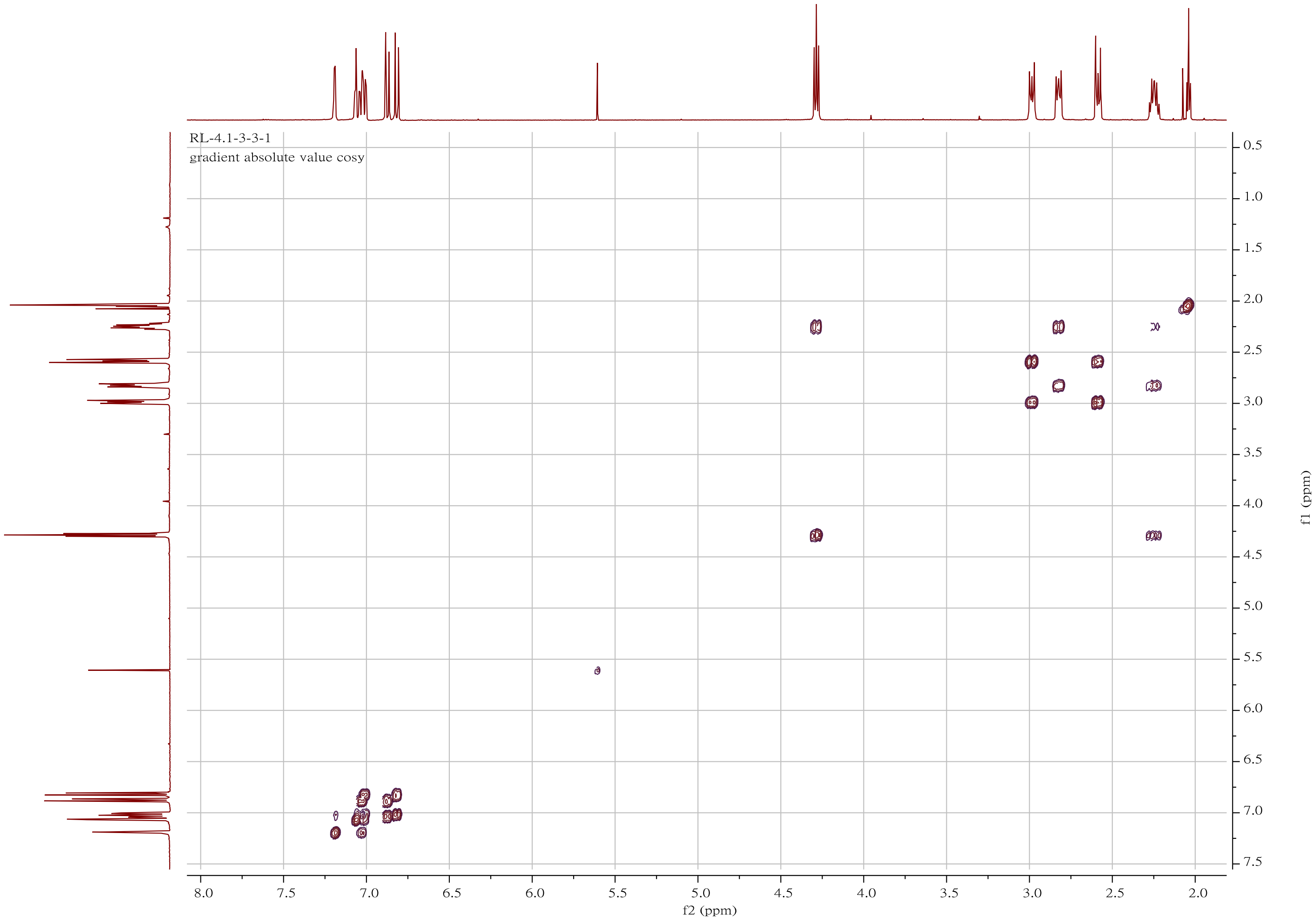

Figure S7: HMBC Spectrum of racelactone A (**1**) in acetone-d_6.

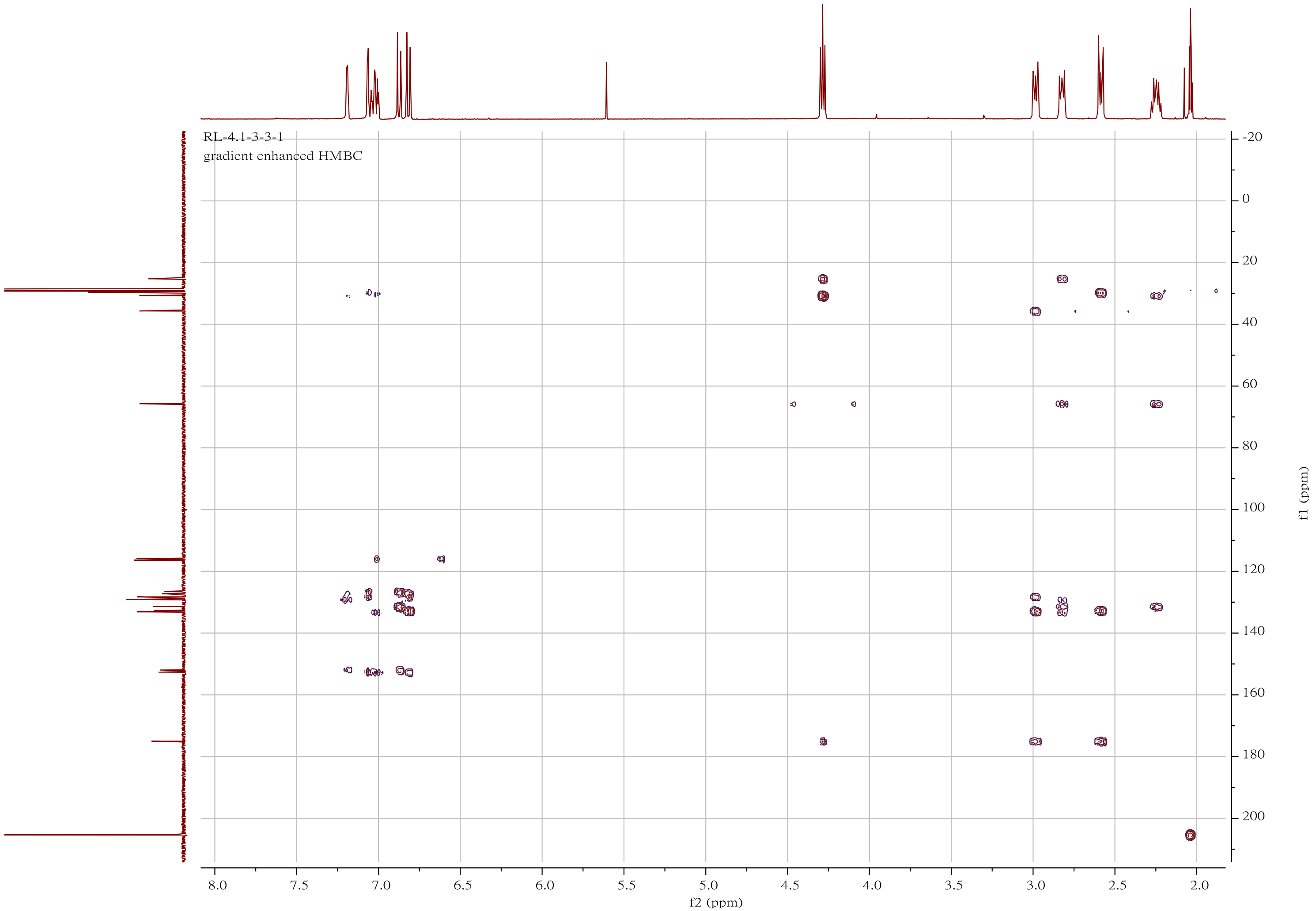

Supplement: Supplementary file 1 [file marinedrugs-16-00404-s001.pdf]
